# Assimilation of socially assistive robots by older adults: an interplay of uses, constraints and outcomes

**DOI:** 10.3389/frobt.2024.1337380

**Published:** 2024-04-05

**Authors:** Oded Zafrani, Galit Nimrod, Maya Krakovski, Shikhar Kumar, Simona Bar-Haim, Yael Edan

**Affiliations:** ^1^ Department of Industrial Engineering and Management, Ben-Gurion University of the Negev, Beer-Sheva, Israel; ^2^ Agricultural Biological Cognitive Initiative, Ben-Gurion University of the Negev, Beer-Sheva, Israel; ^3^ Department of Communication Studies, Ben-Gurion University of the Negev, Beer-Sheva, Israel; ^4^ The Center for Multidisciplinary Research in Aging, Ben-Gurion University of the Negev, Beer-Sheva, Israel; ^5^ Department of Physical Therapy, Ben-Gurion University of the Negev, Beer-Sheva, Israel

**Keywords:** acceptance, aging, assimilation, human-robot interaction, older adults, quality evaluation, socially assistive robots, wellbeing

## Abstract

By supporting autonomy, aging in place, and wellbeing in later life, Socially Assistive Robots are expected to help humanity face the challenges posed by the rapid aging of the world’s population. For the successful acceptance and assimilation of SARs by older adults, it is necessary to understand the factors affecting their Quality Evaluations Previous studies examining Human-Robot Interaction in later life indicated that three aspects shape older adults’ overall QEs of robots: uses, constraints, and outcomes. However, studies were usually limited in duration, focused on acceptance rather than assimilation, and typically explored only one aspect of the interaction. In the present study, we examined uses, constraints, and outcomes simultaneously and over a long period. Nineteen community-dwelling older adults aged 75–97 were given a SAR for physical training for 6 weeks. Their experiences were documented via in-depth interviews conducted before and after the study period, short weekly telephone surveys, and reports produced by the robots. Analysis revealed two distinct groups: (A) The ‘Fans’ - participants who enjoyed using the SAR, attributed added value to it, and experienced a successful assimilation process; and (B) The ‘Skeptics’ - participants who did not like it, negatively evaluated its use, and experienced a disappointing assimilation process. Despite the vast differences between the groups, both reported more positive evaluations of SARs at the end of the study than before it began. Overall, the results indicated that the process of SARs’ assimilation is not homogeneous and provided a profound understanding of the factors shaping older adults’ QE of SARs following actual use. Additionally, the findings demonstrated the theoretical and practical usefulness of a holistic approach in researching older SARs users.

## 1 Introduction

Population aging is expected to be the most significant demographic transformation of the twenty-first century (Morina and Grima, 2021‏). This trend yields numerous social and economic challenges related to health and quality of life in old age (Zhu and Walker, 2021). Embodied technological solutions, and Socially Assistive Robots (SARs) in particular, are expected to play a central role in facing these challenges (Cortellessa et al., 2021; Sorrentino et al., 2022‏). Therefore, it is necessary to understand the factors affecting older adults’ Quality Evaluations (QEs) of SARs.

Previous studies that examined Human-Robot Interaction (HRI) in later life indicated that older adults’ overall QE of robots is shaped by three aspects: their uses, constraints to beneficial use, and use outcomes (Zafrani and Nimrod, 2019). However, previous research has two significant weaknesses: 1) studies typically focused on only one aspect of the interaction between robots and older adults (i.e., examining uses, constraints, and outcomes separately); and 2) most studies were limited in duration, and thus mainly focused on acceptance aspects rather than assimilation. The present study aimed to bridge the gaps in the existing literature. Accordingly, we carried out an assimilation study examining how the QE is shaped following actual interaction with the SAR by a simultaneous exploration of uses, constraints, and outcomes in real-life conditions over a long period.

## 2 Literature review

### 2.1 Quality evaluation of socially assistive robots (SARs)

Technology QE deals with people’s emotions, perceptions, and responses created, derived, and shaped as a result of interaction or anticipated interaction with a system, product, device, or service (Hartson and Pyla, 2012; Lindblom and Andreasson, 2016). The literature on the subject distinguishes between pragmatic and hedonic aspects of evaluation (e.g., Hassenzahl, 2003; Mlekus et al., 2020). The pragmatic aspects of QE relate to the functionality, usability, usefulness, and utility of potential tasks that help users achieve their goals effectively and satisfactorily (da Silva et al., 2019; Hassenzahl and Tractinsky, 2006). Hedonic aspects refer to the users themselves and reflect the emotional benefits they experience when interacting with the technology (Hornbæk and Hertzum, 2017; van de Sand et al., 2020). Positive QEs are necessary to promote acceptance of SARs—a crucial condition for the assimilation process and the realization of the benefits of using robots (e.g., Bensch et al., 2017‏ Naneva et al., 2020).

Acceptance of technology is defined as “the demonstrable willingness within a user group to employ information technology for the tasks it is designed to support” (Dillon and Morris, 1996; Dillon, 2001). The assimilation of technology is defined as the extent to which the use of technology becomes routinized in daily activities (De Mattos and Laurindo, 2017; Purvis et al., 2001). The ability to successfully assimilate new technology depends on users’ absorption or purchase of information and their ability to exploit this information (Kouki et al., 2010). The combination of acceptance and assimilation research suggested that an assimilation pattern can be predicted according to the level of acceptance.

### 2.2 Human-Robot Interaction (HRI) in later life

Research on HRI in later life suggests that three main aspects shape older adults’ overall QE of robots: uses, constraints, and outcomes (Zafrani and Nimrod, 2019). Below are the principal insights concerning each factor.

***Uses.*** This category includes explorations of a) users’ acceptance of new robotics technology, b) processes of adaptation to such technologies, and c) factors affecting user experience. Studies suggested that although older adults were excited about the idea of robots, their acceptance of robotics technologies was ambivalent (Hebesberger et al., 2017; González-González et al., 2021). For example, older adults worried that robotic technologies would replace and even control humans, even though they perceived them as a future extension of existing communications technologies such as the internet and smartphones and expected them to be widely adopted (Liu et al., 2021). In addition, although they believed that robotic systems could support daily activities, older adults said they did not want to use robots (Wang et al., 2017).

Older adults sought robots for object manipulation, physical training, information management, and chores, but preferred humans for leisure activities, information delivery, and personal care (Shen and Wu, 2016; Getson and Nejat, 2021). In this context, they were less receptive to personal assistance such as dressing and bathing but more open to using robotic systems for simple tasks such as managing reminders and communicating (e.g., Robillard and Kabacińska, 2020; Huang and Huang, 2021; Fiorini et al., 2023; Wengefeld et al., 2022).

Although older adults know they are using machines, these users often attribute human qualities to robots and expect them to exhibit human intelligence and behavior (Frennert et al., 2017; Onnasch and Roesler, 2021). In addition, older users expect the robotic technologies to be useful and adaptable to their needs (Olatunji et al., 2020; Kim et al., 2021). A longitudinal study that investigated adaptation to robots demonstrated that giving robots a function in the daily routine of older adults may lead to greater appreciation and approval (De Graaf et al., 2015; Luperto et al., 2022). Furthermore, if users did not ascribe specific functions to robots, they enjoyed the interaction less, gradually lost interest (Torta et al., 2014; Søraa et al., 2022), and eventually returned to their previous habits without robots (Frennert et al., 2017).

***Constraints.*** This category includes explorations of a) antecedent constraints, namely, factors that limit or reduce motivation to use robotic systems, and b) intervening constraints that come between the desire to use robotic systems and the actualization thereof. Among the prominent antecedent constraints were uneasiness with robots (Erel et al., 2021; Gasteiger et al., 2021) and a perception that they had no added value compared to existing modern technologies (Caleb-Solly et al., 2014; Wu et al., 2016; Tonkin, 2021‏). Yet, the stigma associated with using a robot in old age was probably the most dominant antecedent constraint. Healthy older adults perceived the potential robot user as someone frail, lonelier, and more in need of care than them (Pripfl et al., 2016; Bradwell et al., 2021). Therefore, trying to dissociate themselves from these negative stereotypes of old age, they rejected the use of robots.

Major intervening constraints found in the literature were usability and affordability. Concern over robot costs was commonly discussed (Abbott et al., 2019; Koh et al., 2021a). Usability, i.e., the extent to which specified users can use a product or service to achieve specific goals (Nielsen and Madsen, 2012), was of significant worry. Operational difficulties were also an intervening constraint. For example, some studies described users’ dissatisfaction with the robots’ response speed, verbal skills, and comprehension of instructions (Pripfl et al., 2016; Wang et al., 2019).

***Outcomes***. The literature indicated various outcomes from using robots in later life, mostly divided between benefits and risks. Interacting with robots was experienced as enjoyable (Lazar et al., 2016) and cognitively stimulating (Tsardoulias et al., 2017; Louie and Nejat, 2020). It had positive and beneficial effects on older adults’ psychological wellbeing (Henschel et al., 2021), including better communication with friends and family members (Tsardoulias et al., 2017), elevated mood (Khosla et al., 2012), and decreased frustration and stress (Van Patten et al., 2020). Functional benefits often included the robotic technologies’ (e.g., Paro, Nao) contribution to older persons’ quality of life and independence (Tsardoulias et al., 2017; Coşar et al., 2020; Koh et al., 2021; Wang et al., 2021). For example, studies have found that robots support physical exercise and rehabilitation (Avioz-Sarig et al., 2021; Krakovski et al., 2021; Zafrani‏ et al., 2023). In addition, a longitudinal study that used SAR to monitor, assist, and provide social, cognitive, and physical stimulation in elderly homes, provided empirical evidence that SAR can be successfully used for long-term support for older adults (Luperto et al., 2023).

The literature also addressed the risks and negative impacts arising from the use of robots by older people. Regarding psychological risks, concerns related to discomfort or stress resulting from the robot’s appearance, speech, and motion were mainly discussed (Hussain and Zeadally, 2019; Salvini et al., 2021‏). Ethical ramifications such as invasion of privacy and feelings of a loss of control may result from using robots (Caine et al., 2012; Kernaghan, 2014). For example, the presence of cameras and hearing sensors on the robot may cause a feeling of being spied on or under surveillance, which in turn may lead to stress and anxiety (Salvini et al., 2021). Moreover, physical interactions with SARs such as walking support robots (Cifuentes et al., 2014), person-following robots (Olatunji et al., 2020), and mobility robots (Leaman and La, 2017) may create hazards such as accidents or malfunctions (Mansfeld et al., 2018‏).

The holistic approach suggested by Zafrani and Nimrod (2019) postulates that simultaneous exploration of uses, constraints, and outcomes (including both positive and negative effects), rather than focusing on one or two of these issues, may explain how they correlate with one another and provide a broader and more accurate picture of users’ experiences. Moreover, it stresses that extended simultaneous exploration may explain how the HRI and the resulting QE change according to users’ experience, to what extent the interaction is integrated into their daily lives, what factors affect the frequency of use and its benefits, and what constrains beneficial use and leads to decreased frequency or even cessation of use. In contrast to lab experiments, the most common methodology in HRI research, a longitudinal study may help researchers learn and interpret people’s behavior, including how they acquire new knowledge about technology and to what extent they use and retain it over time (King, 2006‏). Furthermore, it provides more accurate insights regarding the assimilation of new technologies into the users’ lives (Cullen, 2018; Nagarajan et al., 2020). It also helps reduce the robot’s novelty and establish its functioning in more realistic situations where the participants are alone with the robot (Yamazaki et al., 2014).

Accordingly, this study applied the holistic approach (Zafrani and Nimrod, 2019) to answer the following questions:

1. What are the uses, constraints, and outcomes that older adults experience while assimilating a SAR into their lives?
2. Do the uses, constraints, and outcomes change during the assimilation period? If so, how?
3. How do older adults’ experiences with a SAR over a long period and in real-life conditions shape their QE of that SAR in particular and of SARs in general?

## 3 Methods

### 3.1 The robotic system

**FIGURE 1:**
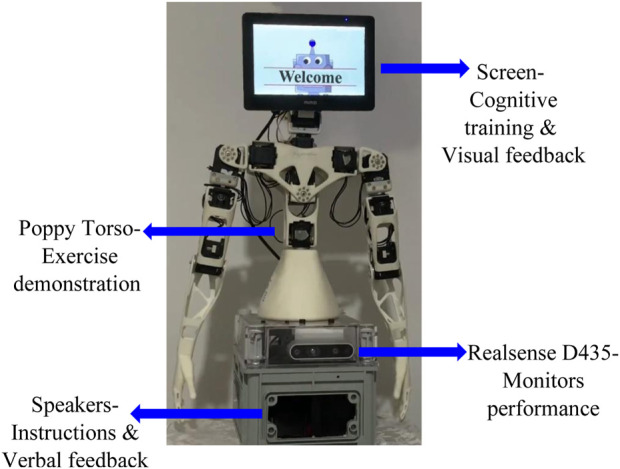
‘Gymmy’—personal training robot.

To answer the research questions, we used a SAR developed in our lab (Figure 1). This SAR—named ‘Gymmy’ to elicit the associations of the word “gym”—was designed to serve as a personal trainer that would support aging individuals’ physical activity. During the training sessions, the robot demonstrates a series of physical exercises and follows their execution. The users perform the exercise with the robot, and a camera monitors their movements. If needed, the robot corrects the execution. In addition, during the physical training, cognitive training sessions, such as memory and thinking exercises, are randomly presented to the users. Furthermore, the system offers users relaxation exercises to release stress and relieve pressure, according to Jacobson’s relaxation technique (Jacobson, 1938). The system includes a humanoid mechanical-looking robot (Poppy Torso) and a computer system (NUC mini-PC) to demonstrate the exercises, an RGB-D (red-green-blue-depth) camera to monitor the user’s performance (Intel RealSense™ D435), speakers and a touch screen for instructions and feedback (for additional information about the development of the system, see Krakovski et al., 2021; Zafrani‏ et al., 2023, or watch https://www.youtube.com/watch?v=zQ4T1NhS25Q).

This robotic system we developed was used in our previous studies where each study had its own novelty. In Krakovski et al. (2021) we presented the development of “Gymmy”, and conducted 1-day experiments in home environments to examine the effect of users’ characteristics (age, gender, education and attitude toward robots), on the acceptance of the robotic trainer. In Zafrani et al. (2023) we conducted an online survey to explore the anticipated interaction through video viewing of a SAR (Gymmy). The novelty of this work is that we investigated how the QE is shaped following actual interaction with a SAR (Gymmy) by a simultaneous exploration of uses, constraints, and outcomes in real-life conditions over a long period.

#### 3.1.1 Physical exercises

**FIGURE 2:**
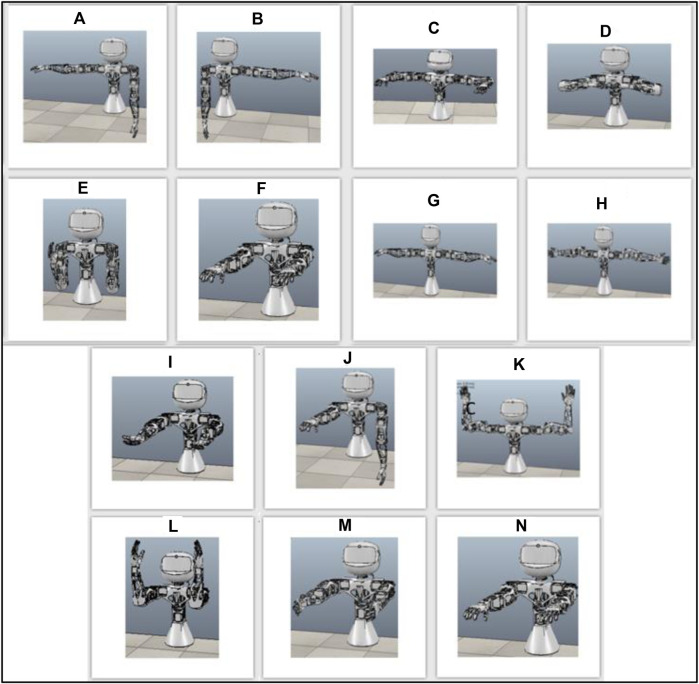
**(A, B)**- raise arms horizontally separately, **(C)**-raise arms and bend elbows forward 90, **(D)**-raise arms and bend elbows, **(E)**-bend elbows, **(F)**- raise arms forward static **(G)**-raise arms horizon-tally, **(H)**-raise arms horizontally and turn hands, **(I)**-raise arms forward and turn hands, **(J)**-raise arms forward separately, **(K)**-raise arms 90 and up, **(L)**-open and close arms 90, **(M)**-raise arms forward and to sides, **(N)**- raise arms forward.

Gymmy’s physical training focused on exercises for the upper body, which matched the functionality of the Poppy robot’s torso version. These exercises improve muscle strength and help older adults maintain their independence and perform daily activities such as lifting objects. A total of 14 physical training exercises were developed (Avioz-Sarig, 2019; Krakovsky et al., 2021; Figure 2) according to the recommendations of the National Institute on Aging (NIH; https://go4life.nia.nih.gov/exercise-type/strength/retrieved July 2019).

#### 3.1.2 Cognitive exercises

Gymmy’s cognitive training was designed to address different aspects of memory, processing speed, and concentration, which are crucial for older adults’ ability to live independently (Eggenberger et al., 2015; Arora, 2021). Three cognitive games were randomly integrated during the physical training sessions. These games were chosen based on the literature (e.g., Nacke et al., 2009; Ezzati et al., 2016). Each game started with instructions, and then, using the touch screen, users confirmed that they were ready to start the game.

#### 3.1.3 Relaxation exercises

Designed according to Jacobson’s relaxation technique (Jacobson, 1938), Gymmy’s relaxation exercises were provided to release stress and relieve pressure. This tool is essential for older adults’ wellbeing (Rudnik et al., 2021‏‏‏) and allows them to perform relaxation exercises for three muscle systems: arms, neck, and face.

### 3.2 Participants and sample description

Participants were recruited through mailing lists of retirees, public announcements, and snowball sampling. Criteria for participation were age 75 years and over, namely, the “old-old” category (Kubota et al., 2012; Boot et al., 2020) and independent living. Nineteen community-dwelling older adults (age ranged between 75 and 97, mean = 81.05, SD = 6.19) who resided in cities (N = 12) or ‘kibbutzim’ (i.e., a rural community defined by its commitment to mutual aid, community living, and social justice, N = 7) in the southern part of Israel participated in this study. Nine participants were men (47%), and 10 were women (53%). Nine participants were married, eight were widows, one was in a permanent relationship, and one was divorced. All participants had children (range = two to five, mean = 3.31, SD = 1.05). The majority had secondary education, and eight had post-secondary education. Most participants were not born in Israel (N = 17), and 14 were secular. All participants were retired except for one who still worked full-time.

### 3.3 Data collection

For all study participants, a unit of Gymmy was installed in their homes for 6 weeks. In-depth semi-structured interviews were conducted with each participant at their homes before and after the study. The first session with the participants opened with oral and written explanations about the study. After signing a consent form, the participants filled out a demographic, sociodemographic, and health background questionnaire (Appendix A). Then, each participant was given detailed explanations about Gymmy, watched a video that presented its functions, and was interviewed. In these in-depth interviews (Appendix B), participants shared their biographical and occupational backgrounds, daily routines, and Information and Communication Technology (ICT) use. The main goal of these interviews was to explore their expectations of SARs in general and from Gymmy in particular. Therefore, they were also asked questions about the advantages, disadvantages, risks, and benefits that they believed existed in SARs and questions that specifically focused on their expectations from Gymmy.

To prevent participants’ exhaustion, Gymmy’s installation was done several days after the preliminary interview. This session included installation (15–20 min), guidance (25–30 min), and demonstration of full training with Gymmy (about 15 min). It was explained to the participants that the use of the robot is according to their will, only when they want, and in a proactive way, that is, if the user wants to use Gymmy, he/she has to press the dedicated power button. In addition, it was explained to the participants that communication with the robot is multimodal, based on speech, movement (hand waving), and touch (Krakovski et al., 2021). Throughout the study period, participants were offered unlimited technical support. After 6 weeks, at the end of the study period, concluding interviews were conducted with the participants (Appendix C). These interviews examined their overall experience with Gymmy vis-à-vis their initial expectations. Thus, they were asked direct questions about the frequency of use, difficulties of use, and the advantages, disadvantages, risks, and benefits that they thought existed in using Gymmy. All interviews were audio-recorded and transcribed verbatim.

In addition to the in-depth interviews, a short weekly telephone survey (Appendix D) was conducted with study participants. They were asked to rate their level of satisfaction with Gymmy and the extent to which they faced operational problems using a five-point Likert scale ranging from 1 (“not at all”) to 5 (“a lot”). Lastly, the robot automatically produced usage reports, which included accurate information about the frequency and usage dates. Due to a limited number of Gymmy units, the data were collected in five cycles, with four-five participants in each cycle.

### 3.4 Data analysis

Analysis began with the qualitative data collected in the interviews and followed Miles and Huberman’s (1994) strategies of noting patterns, contrasting and comparing, and clustering. This phase started with within-case analysis and proceeded to cross-case analysis. Hence, each participant’s pre-use and concluding interviews were first independently coded. Then, they were compared with other participants’ interviews to elicit similarities, contrasts, and overlaps in relation to the codes found. The coding method was inductive, using open coding and axial coding techniques to make connections and group the codes into categories according to content (e.g., risks, benefits). Applying this method allowed the findings to emerge from the text without any preexisting concepts. The analysis was performed by the first two authors and then meticulously reviewed by the other authors. The authors discussed and re-analyzed unclear codes and discrepancies to strengthen validity (Hammersley, 1992). Accordingly, the meta matrix was reorganized several times and expanded with new categories and codes.

As the qualitative analysis revealed two distinct groups of study participants representing different assimilation patterns, the quantitative data (i.e., the usage reports) were used to further explore the differences between the groups. Accordingly, the next chapter will combine quantitative and qualitative findings. Pseudonyms were used to guarantee anonymity.

## 4 Findings

The analysis revealed two distinct assimilation patterns: (A) The ‘Fans’ - participants who enjoyed using Gymmy very much, trusted it, attributed added value to it, and experienced a successful assimilation process, and (B) The ‘Skeptics’ - participants who did not like Gymmy, experienced a disappointing assimilation process, and therefore expressed no interest in using it after the research period was over. The identified groups differed in their background, attitudes towards robots before and after using Gymmy, and actual use experience. The following sections describe in detail the process of assimilation of the two groups, including their characteristics, attitudes, and experiences.

### 4.1 The ‘Fans’

#### 4.1.1 Personal background

The average age of the nine participants who liked Gymmy ​ranged between 75 and 97 years, with a mean age of 82.88 years (SD = 7.57), and their mean number of years of education was 12.1 (SD = 2.89). Most (N = 7) of these participants came to the study without experience or knowledge of robotics. Before the period of use of Gymmy, six participants in this group performed only basic physical activity several times a week, such as walking, two participants did not exercise at all, and one participant exercised in a gym. In addition, their use of various media focused mainly on traditional uses such as making phone calls and watching television.

#### 4.1.2 Attitudes towards SARs before use

Seven participants from the ‘Fans’ group came to the study without actual attitudes towards SARs because, as mentioned, they had no previous experience or knowledge of SARs. Yet, all participants in this group showed great curiosity and a desire to experience the use of robotics. Helen (W, 86, Widow) explained: “Even at my age, I still want to learn new things … I have curiosity, it is always good to know more things, and it is just interesting, to keep evolving, not to stand still,” and Daphna (W, 97, Widow) shared: “I agreed to participate in the study because I am interested in new things, I am very interested in it.”

#### 4.1.3 Actual use experience

**FIGURE 3:**
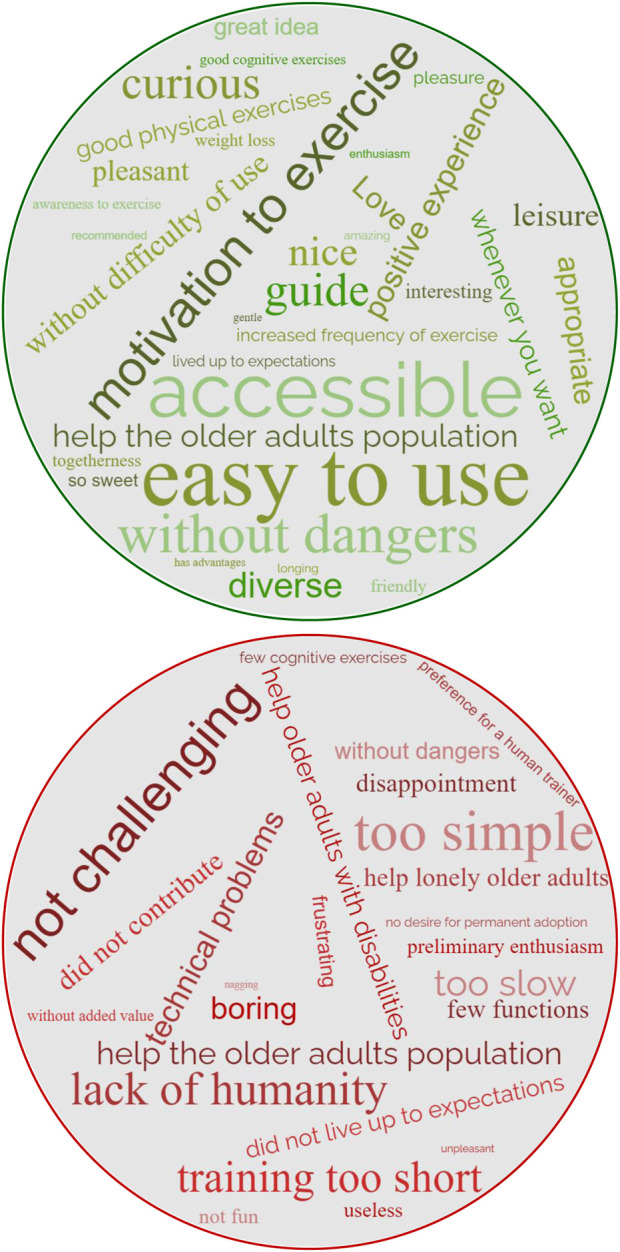
Word clouds that describe the actual use experience of the ‘Fans’ (green) and the ‘Skeptics’ (red). Note: The bigger the word in the word cloud, the more frequently it appeared in the interviews.

**FIGURE 4:**
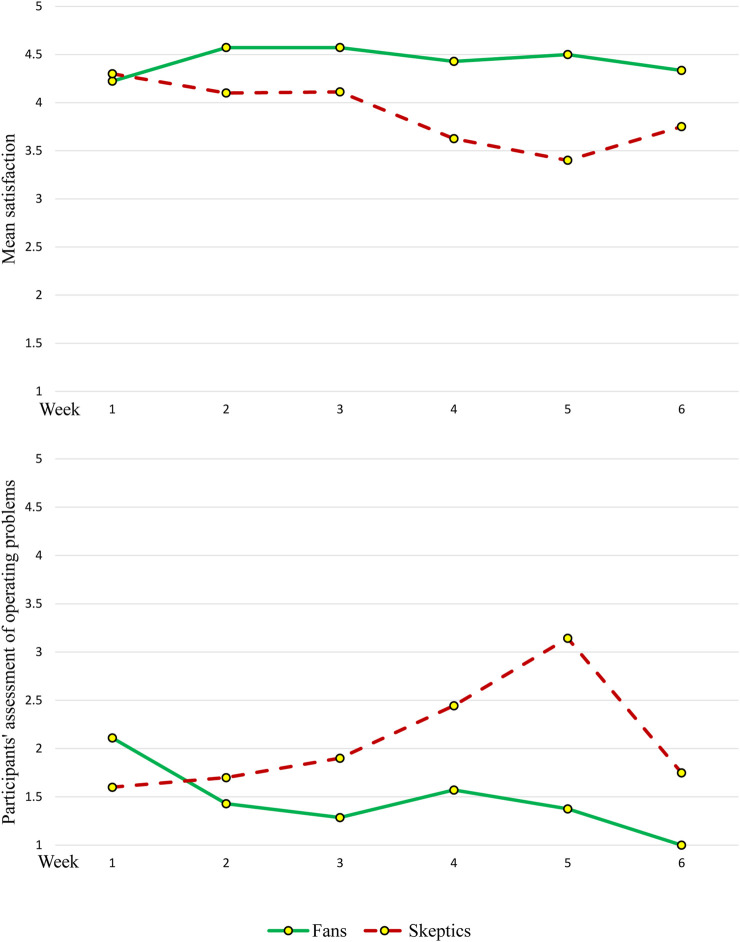
Six-weeks self-report use trends among the two groups of study participants.

**FIGURE 5:**
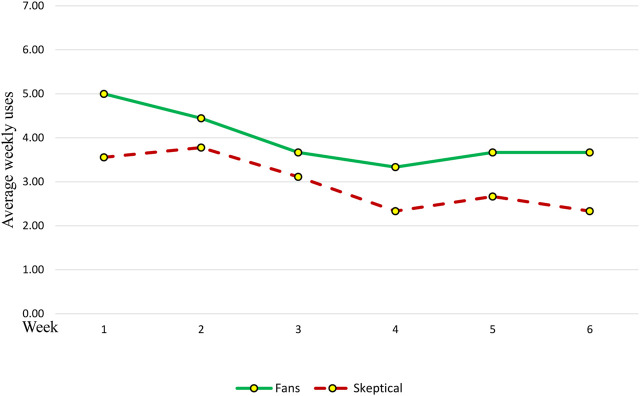
Average weekly uses among the two types of study participants according to the SAR’s reports.

Participants in the ‘Fans’ group loved Gymmy very much (Figure 3; Figure 4), trusted it, appreciated its pragmatic and hedonic aspects, its ‘unique use characteristics’ and its ‘advantages,’ used it regularly throughout the study period (Figure 5), experienced ‘positive evaluations’ towards it, and even reported ‘positive outcomes’ from its use. Moreover, this group of participants directly connected Gymmy’s ‘unique use characteristics’ to the ‘advantages’ they found in it. That is, its unique use characteristics are its advantages, and its advantages lie in the characteristics of its unique use. Its unique uses characteristics included pragmatic aspects)*easy to operate, convenient to use,* and *provides guidance and demonstration*) and a hedonic aspect (*has humanity*).

A powerful influence on the participants’ experience was that Gymmy was *easy to operate*. The participants did not experience any use problems or difficulties during the study period (Figure 3). Miley (W, 75, Married) indicated that using it was “super easy … there is no need to have Einstein’s intelligence to operate it.” Another factor that played an essential role in the participants’ experience was Gymmy’s *convenience of use*. This factor contained two interrelated characteristics, Gymmy’s *accessibility* and *availability*. Gymmy was placed in the most accessible place for the participants, i.e., in their homes. Therefore, they could exercise “without leaving their home.” Additionally, the fact that Gymmy was always available allowed them to use it whenever they wanted. Sami (M, 86, Married), for example, pointed out that “compared to a human trainer, who will not come to your home whenever you want, Gymmy is always available to you.”

Another unique characteristic of Gymmy noted by these participants was that it *provided guidance and demonstration* regarding how to perform the exercises. As Helen (W, 86, Widow) avowed: “I really like getting guidance on what to do.” Finally, Gymmy’s *humanity* was frequently discussed when participants regarded it as a human presence at home. For example, Paula (W, 91, Divorced) always *anthropomorphized* Gymmy when she talked about training with it and described the routine of her encounters with Gymmy as a human routine for all intents and purposes: “Every time I met him, I said hello to him, I made the movements with him, and it answered me very well,” and explained: “I did the exercises according to what he said.”

The ‘positive evaluations’ described by the ‘Fans’ were for Gymmy itself, the experience of its use, and the functions it offered. First, the participants chose to describe it with many affectionate adjectives such as “so sweet,” “amazing,” “very nice,” “pleasant,” “gentle,” and “friendly.” In addition, they praised it with compliments for being an “excellent idea,” “interesting,” and “intriguing.” Moreover, they indicated that Gymmy *suited their daily needs* and provided a *good physical and cognitive training level.* They experienced *enthusiasm and enjoyment* and had a *positive experience* thanks to it.

Participants described Gymmy’s *suitability for their daily needs* in a variety of superlatives and explained that it “suited me exactly,” “came to me at the right time,” and was “exactly what I needed.” Helen (W, 86, Widow) detailed that “Gymmy allows me to do exactly what I can, it suits me very well … it keeps me busy in a pleasant way.” This adequacy between the participants’ needs and the use of Gymmy was made possible thanks to the fact that Gymmy provided the ‘Fans’ with *a good level of physical and cognitive training*. From the point of view of physical training, participants in this group noted that they experienced “diversity in the type of physical activity,” that “Gymmy’s movements were nice,” and that “the number of repetitions was good.” From a cognitive training perspective, participants reported that the assignments were “good,” “clear,” and “without problems.”

In the concluding interview, when these participants were asked to describe the period of their use of Gymmy, they pointed out that they experienced *enthusiasm and enjoyment* and had *a positive experience* thanks to it. *Enthusiasm and enjoyment* referred to positive feelings that Gymmy and its features aroused among the participants during the study period. For example, Daphna (W, 97, Widow) shared that “moving the muscles and making an effort is the best thing I can do.”

The feelings of pleasure experienced during the study period directly affected the participants’ sense at the end of the study period, which they defined as a *positive experience*. For example, Alex (W, 79) remembered longingly: “Gymmy made me smile in the morning when it said good morning, my name is Gymmy … I approached it happily; it was pleasant and comfortable for me. It was a good experience. I’m a little sad because Gymmy is leaving; I really like it.”

As a result of the successful use they experienced, the ‘Fans’ obtained several interrelated ‘positive outcomes.’ They reported that Gymmy strengthened their *awareness and motivation to exercise* and increased their *exercising frequency.* Gymmy’s presence in the participants’ homes contributed to their general *exercise awareness*. As Nina (W, 77, Widow) shared: “It is really a problem to move the body … I was more aware that I had to get up from the computer.” In addition to awareness, they noted that Gymmy *motivated participants to exercise more*. Participants explained that Gymmy was actually like a training partner, one who “moves with me,” “spurred,” “encouraged,” and “pushed me to exercise at home.”

As the awareness and motivation to exercise increased, so did *the frequency with which participants exercised*. That is, the awareness, motivation, and the fact that Gymmy was accessible and available led to an increase in the participants’ total physical activity time during the study period. When asked by the interviewer in the concluding interview if the robot made them perform more physical activity, most of the participants in this group answered ‘yes,’ and shared a variety of positive responses, such as “Of course it added.”—Luca (M, 79, Married); “Certainly, definitely, now more.”—Helen (W, 86, Widow).

#### 4.1.4 Attitudes towards SARs after use

The use of Gymmy led to a positive overall evaluation of SARs among Gymmy’s ‘Fans’ and positively influenced their perceptions. These participants shared in the concluding interviews that they believe that using SARs “can undoubtedly” *help the older adults.* “For older adults? For sure! A thousand percent, it will make a great contribution to a person,” stated Tom (M, 76, Widow). Similarly, Luca (M, 79, Married) highlighted that “robotics can benefit older people in many areas … it can save time, money … it can only be beneficial.” Further evidence that the use of Gymmy positively affected the perception and evaluation of SARs among this group of participants stemmed from the question about the dangers and risks of robots asked during the concluding interviews. Without exception, all the participants in this group indicated that “there are no risks at all,” only “positive things.”

### 4.2 The ‘skeptics’

#### 4.2.1 Personal background

The average age of the ten participants in the ‘Skeptics’ group ranged from 75 to 86 years, with a mean age of 79.4 years (SD = 4.41). This group’s mean number of years of education was 13.5 (SD = 2.28). Hence, they were somewhat younger and more educated than the ‘Fans.’ Compared to the first group, most (N = 8) of the ‘Skeptics’ also came to the study with knowledge, previous experience, and familiarity with the world of robotics. This may explain why they expressed some skepticism during the preliminary in-depth interviews and mentioned many disadvantages that they believed existed in using SARs and a variety of potential risks that may result from this use. Most of the ‘Skeptics’’ exercise habits before the study were extensive and diverse. Finally, the media use of all participants in this group included both basic and advanced uses of ICT.

#### 4.2.2 Attitudes towards SARs before use

Eight participants in this group joined the study with previous knowledge and experience with SARs. The accumulated knowledge came from “books,” “movies,” and “lectures on the subject,” and as a result of experiences shared with them by acquaintances. It seemed that their early familiarity and being ‘knowledgeable’ about robotics made them come to the study with a sense of skepticism. Michael (M, 82, Married), for example, shared: “We must find a way to balance the wisdom of the robots so that they cannot do everything … otherwise we will close the hospitals, kill the people, and use only robots,” and summarized: “I hope I can get along with Gymmy.”

In accordance with these sentiments, they described several disadvantages that characterize the use of SARs and risks that they believed may be caused by this use. The ‘disadvantages’ included: *robots are not a substitute for a human,* and *robots depend on the person who programs them*. *Robots are not a substitute for a human* was frequently discussed when comparing the interaction with a robot to that with a human. Participants repeatedly emphasized that “robots lack human contact.” Maggie (W, 77, Widow), for example, explained that: “There is no substitute for a look in the eyes and a hug, for laughing together, for all the things that a human being gives.” In addition, the ‘Skeptics’ explained that SARs could not be a worthy substitute for humans as they cannot experience and express emotions whereas “human and emotion cannot be separated,” as highlighted by Clara (W, 75, Married). The participants noted that, like other modern technologies, *robots depend on the person who programs them* for better or worse. Therefore, participants said they hope the programmer has “lofty goals and aims to help users.”

Along with the disadvantages, participants also noted three potential ‘risks’ that may result from using SARs: *Invasion of privacy, impairment of independence in daily functioning,* and *risk of being replaced by robots*. *Invasion of privacy* was discussed in terms of informational privacy. Participants shared their concerns about their understanding of how the information shared with the robot is processed and used (or misused). For example, Nathan (M, 75, Married) said: “Actually, I have no idea what the robot is doing, and I am afraid there will be ​an invasion of my private life.”

The second risk discussed by the participants was *impairment of independence in daily functioning,* both physically and cognitively. From a physical perspective, this risk was derived from the participants’ concern about being replaced by robots in daily activities at home. The participants claimed that robots might threaten their autonomy by replacing them in home tasks they should perform by themselves. Sofie (W, 77, Married), for example, clarified: “I get along great without it, I leave it for the future.” From a cognitive perspective, the fact that robots are an extremely ‘intelligent’ technology that can efficiently perform cognitive actions was a potential risk, according to participants’ perceptions. They claimed that the robots may threaten users’ cognitive abilities in knowledge tasks they can perform independently, as Gabriel (M, 80, Married) shared: “We will think less, and the robots will think for us.”

The *risk of being replaced by robots* was discussed in the employment context. “This is a pretty difficult problem for the world … if robots come into our lives … people will lose their jobs etc.,” explained Sarah (W, 83, Widow). Michael (M, 82, Married) even portrayed the future as an apocalyptic scenario and explained: “Robots pose a danger of replacing people … you will have no job, and you will have nothing.”

#### 4.2.3 Actual use experience

The ‘Skeptics’ first days of using Gymmy can be described as a success. The participants learned how to use it, were satisfied with it, and experienced enthusiasm. However, the excitement was only temporary and preliminary, a finding reflected in the usage data produced by Gymmy (Figure 5) and in the participants’ words. For example, Daniel (M, 75, Married) explained: “At first it was nice … it was a new experience to play with a robot.” In fact, the initial enthusiasm cooled down and turned into disappointment, which was reflected in the participants’ negative evaluations (Figure 3). These evaluations were due to several pragmatic factors (*too simple and not challenging enough, technical problems, too slow,* and *few activities*) and a result of one non-hedonic factor (*lack of humanity*).

The biggest disappointment with Gymmy among the ‘Skeptics’ was that the participants perceived its use as *too simple and not challenging*, and even boring. As Clara (W, 75, Married) said: “I was expecting something more challenging, but it was not … it is too simple for my abilities.” This was true for physical training, cognitive training, and relaxation exercises. This simplicity and lack of challenge made the participants feel that the use of Gymmy was “somewhat frustrating,” “nagging,” and “not fun.”

Another major issue noted by the participants was *technical problems*. The ‘Skeptics’ showed less patience for technical faults, a tendency clearly reflected in their weekly reports (Figure 4) and directly affected their sense of disappointment. “The difficulty is that there were some exercises where the robot did not catch my movements,” noted Nathan (M, 75, Married). *Too slow* referred to the time it took Gymmy to turn on and perform the exercises and the waiting time between them. Maggie (W, 77, Widow), for example, noted that “it takes a long time for Gymmy to wake up.” In addition, Daniel (M, 75, Married) said: “The exercises should be much faster … more vigorous.” Nathan (M, 75, Married) commented that the waiting time between exercises “is very long, so I did a run-in-place activity meanwhile.”

*Few activities* were discussed in the context of two aspects: First, regarding a too-small pool of physical exercises, and second, regarding the fact that the physical training was for the upper limbs only. Regarding the first aspect, several participants expressed disappointment that the physical training time was too short. “I just started, and immediately it was over,” indicated Arik (M, 86, Widow). In terms of the second aspect, participants perceived the physical training for upper limbs only as a disadvantage, were disappointed by this, and hoped for additional activities that would allow them to train their lower body. For example, Clara (W, 75, Married) mentioned that “the lower part of the body did not participate … it was completely missing.”

*Lack of humanity* as a factor that caused a negative evaluation among the ‘Skeptics’ stemmed from the fact that they expected the communication with Gymmy to be as similar as possible to human-human interaction. Therefore, when expectations did not match reality, most experienced disappointment. Daniel (M, 75, Married), for example, described that “it would have been perfect if it had said, ‘Come on, Daniel, time to practice.’” In almost the same way, Maggie (W, 77, Widow) explained: “The only thing I wanted was that the robot would talk to me … but it did not talk.”

Following all the factors listed above, most ‘Skeptics’ felt that Gymmy “does not provide any added value” and that “there is no novelty in it.” Accordingly, Gymmy “did not live up to expectations,” and they felt “no desire for permanent adoption.” For instance, Clara (W, 75, Married) concluded sarcastically: “There was nothing special here; I did not shed a tear because Gymmy was leaving.”

Notwithstanding the disappointment these users experienced, and despite their disinterest in adopting ‘Gymmy’ permanently, their attitude towards it after use was that it had a ‘potential for others.’ Although Gymmy was less relevant for them, they believed that it could certainly help *lonely older adults* and *older adults with disabilities.* Help for *lonely older adults* referred to the perception that Gymmy can act as a kind of companion or friend. As Gabriel (M, 80, Married) explained: “This robot can be an advantage for lonely persons … Gymmy can definitely guide them and make them exercise.” Another perception that participants shared about Gymmy was that it could help *older adults with disabilities.* The fact that Gymmy is placed at home and can be used at any time allows “people who cannot leave their homes” or “people who are confined to a wheelchair” to exercise.

#### 4.2.4 Attitudes towards SARs after use

This group’s skepticism in the opening interviews seemed to impact their evaluation of robots after the study period. Nevertheless, the disadvantages and risks that this group of users associated with SARs appeared to be somewhat moderated by their experiences with Gymmy. Accordingly, their evaluations of SARs at the end of the study were, in most cases, a little more positive than they were before it began. For example, Maggie (W, 77, Widow) explained before the study period that “robots have advantages and disadvantages, but you have to get used to it, it is something so new.” At the end of the study, when asked if, after using Gymmy, she is more open to future experiences with robots, she replied: “I think so … I’m open to that.” Not all Skeptics, however, demonstrated greater openness and better evaluations of robots after the study. For example, Sofie (W, 77, Married), who said in the opening interview that she was doing fine without a robot, maintained the same mindset after the study was over: “I never had any issues that required the use of robots.”

Notwithstanding the differences mentioned above, all ‘Skeptics’ noted ‘potential benefits’ they believed could result from using SARs. The benefits included *assistance to the older population in general* and *to lonely older adults and older adults with disabilities in particular.* Similar to the ‘Fans,’ but less decisively, the ‘Skeptics’ also believed that using SARs could help the older population. At the same time, their focus was on sub-populations within the older population. Like their attitude towards the specific potential of Gymmy, they believed that SARs could help mostly *lonely older adults* and *older adults with disabilities.* Finally, when the ‘Skeptics’ were asked if, after using ‘Gymmy,’ they thought SARs could be dangerous, apart from two participants, all the others stated that they were “not afraid of using them” nor did they think they might be hazardous.

## 5 Discussion

Following the users’ experiences in real-life conditions and over time made it possible to identify two distinct assimilation patterns in terms of uses, constraints (both antecedent and intervening), and outcomes (both benefits and disappointments). The two patterns suggested that the process of SARs’ assimilation is not homogeneous and provided a more profound understanding of the factors affecting older adults’ QE of SARs following actual use. Below is a discussion of the two assimilation patterns vis-à-vis the three main topics explored regarding HRI in later life, namely: uses, constraints, and outcomes (Zafrani and Nimrod, 2019).

***Uses***. In the present study, the two groups of users reported a completely different use experience. Whereas the ‘Fans’ experienced Gymmy as easy and convenient to operate and use, the ‘Skeptics’ experienced it as too simple, unchallenging, and boring. A possible explanation for this gap in the user experience lies in the participants’ exercise habits before the study. Gymmy provided the ‘Fans’ with a new value-added function to their daily routines, i.e., physical activity, which they either did not engage in at all or did so to a very limited extent prior to the study. In contrast, this function was adequately implemented in the Skeptics’ daily routines. Accordingly, they did not attribute added value to the use of Gymmy, which, in turn, led to decreased intensity of use.

This explanation is consistent with previous research suggesting that user attributes significantly affect the user experience (e.g., Morillo-Mendez et al., 2021). It also echoes studies demonstrating that older adults expect robots to be tailored to their needs (Tsardoulias et al., 2017; Karkovsky et al., 2021). If they cannot ascribe new valuable functions to the robot, they will evaluate the interaction with it less favorably and eventually abandon its use (Frennert et al., 2017).

The present study’s findings also supported previous research, according to which direct experience with SARs promotes acceptance (Shen and Wu, 2016). Most of the participants in the ‘Fans’ group came to the study without any explicit attitudes towards SARs because, as mentioned, they had had no prior familiarity with the field of robotics. However, at the end of the study period, they developed positive attitudes towards SARs in general. Moreover, despite the disappointment with Gymmy, the direct interaction with it reduced the ambivalence towards SARs among the ‘Skeptics.’ These participants came to the study with firm negative attitudes towards SARs and mentioned a variety of disadvantages and risks that they believed were associated with their use. Nevertheless, their overall evaluations of SARs at the end of the study period were more positive than beforehand.

***Constraints.*** The discussion of constraints refers solely to the ‘Skeptics,’ as the ‘Fans’ hardly reported any constraints. The gap between the ‘Fans’ and the ‘Skeptics’ resulted not only from the differences in exercise habits, but also from a most significant antecedent constraint found among the ‘Skeptics.’ As stated above, unlike the ‘Fans,’ most of the participants in the ‘Skeptics’ group joined the study with previous attitudes towards SARs, which were constructed by contents to which various media exposed them. In such contents, robots are often demonized and presented as attempting to take over the world and replace humans (e.g., the Terminator; Bartneck et al., 2007). These negative connotations, in turn, can trigger negative attitudes and emotions toward robots (Lee et al., 2012).

This gap may also stem from the characteristics of the sample. As mentioned above, the ‘Skeptics’ were somewhat more educated than the ‘Fans.’ It is thus reasonable to argue that older adults with a higher level of education and prior knowledge of robotic technologies would be characterized by a more realistic perception and greater awareness of their shortcomings. Simultaneously, the education variable may also affect the degree of exposure to various media content (Huffman, 2018‏) and the ability to acquire and absorb information and content from modern technologies (Simoni et al., 2016). Therefore, the initial negative attitudes of the ‘Skeptics’ could result from exposure to content in the various media, from being more educated, or from the correlation between these variables.

Another antecedent constraint found among the ‘Skeptics’ came from the stigma associated with using a robot in old age (Neven, 2010; Bradwell et al., 2021), which is one of the most dominant constraints found in the literature on HRI in later life (Zafrani and Nimrod, 2019). The ‘Skeptics’ tended to perceive the prospective robot user as a much older person who needs substantial support with everyday tasks. This negative perception limited the Skeptics’ motivation to use SARs even before the study and may have helped them dissociate themselves from ageist stereotypes.

Intervening constraints found were technical problems and slow operation. The ‘Skeptics’ showed less patience for these issues than the ‘Fans’—a finding reflected in the concluding interviews that seemed to affect their evaluations of Gymmy. These findings support previous literature indicating that usability, including various operative difficulties in robot performance, constitutes a significant intervening constraint (Pripfl et al., 2016; Wang et al., 2019) that leads to dissatisfaction and negative QE (De Graaf et al., 2015; Wang et al., 2019).

***Outcomes.*** The discussion of outcomes is divided into negative and positive outcomes (i.e., benefits). Similar to the constraints, the entire negative outcomes category was relevant only to the ‘Skeptics.’ These outcomes were reflected in the fact that throughout most of the study period, they experienced a host of negative sentiments (dissatisfaction, boredom, frustration, resentment, and lack of enjoyment, patience, and interest) and were overall disappointed with using Gymmy.

Besides describing the negative outcomes, the participants addressed the benefits gained from using Gymmy and the potential benefits that they believed existed in SARs following the study. Here, the division between the ‘Fans’ and the ‘Skeptics’ was noteworthy: Whereas the benefits mentioned by the ‘Fans’ were directed at themselves, the benefits described by the ‘Skeptics’ were directed at others. The positive feelings reported by the ‘Fans’ echoed previous research on HRI in later life, suggesting that interacting with robots was experienced by older adults as an enjoyable activity (De Graaf et al., 2015; Lazar et al., 2016) that had positive effects on their mood (McGlynn et al., 2017) and wellbeing (Henschel et al., 2021). The ‘Fans’ noted additional benefits related to the central function that Gymmy provided - physical training. They reported that Gymmy raised their awareness and motivation to exercise and increased their exercise frequency.

Among the ‘Skeptics,’ Gymmy’s specific potential benefits were the same as the potential benefits of SARs in general. They believed that Gymmy, like any SAR, could assist the older population, especially lonely and frail older adults. It can be assumed that this perception was related to the antecedent constraint mentioned above regarding the stigma of this group associated with using a robot in old age.

Overall, the application of simultaneous exploration of uses, constraints, and outcomes over time and in real-life conditions explained how these topics correlate with one another and presented a broader and more accurate picture of factors shaping older adults’ QE of SARs. Specifically, this exploration explained how the QE may vary according to the assimilation pattern, the factors affecting the use and the benefits gained, and the constraints to beneficial use. Moreover, this study showed an inevitable connection between these three topics, as the interchange between uses and constraints seems to influence outcomes. Accordingly, it is suggested that future research should apply a similar approach.

The findings also suggest two integrative practical recommendations for improving SARs’ evaluation by older adults. First, to promote a more positive QE, acceptance, and successful assimilation process, developers and designers of SARs for older adults are advised to consider the needs of older adults and take steps to reduce their fears, concerns, and sense of inconvenience about robots. The features that make the robots pleasant to use should be stressed in all educational and marketing communication targeting older people. Moreover, a proper training session on the SAR and its functions should be provided, during which participants are given relevant information and allowed to ask questions to remove their doubts and fears. Second, it is essential to invest efforts in developing and designing SARs that are adjustable to the user’s needs, functional, convenient, simple, provide added value, easy to use, and have unique features such as a multi-modal communication (Kirby et al., 2009; Krakovski, 2022).

## 6 Limitations and future research

Despite its many strengths, this study has several limitations that should be acknowledged. First of all, the study was limited to one specific SAR designed for a particular purpose (physical training), and thus the findings cannot be generalized to other robotic systems. Using a SAR intended for another purpose or using a multi-purpose SAR might have yielded different results. In addition, most of the participants in the study were healthy older adults without physical or cognitive impairments. Therefore, we cannot generalize its findings to frail older adults. Finally, the study was conducted during the COVID-19 pandemic, which may have affected the participants’ exercise patterns, general mood, and assessments.

Future research should expand the present study to explore factors affecting QE of SARs among additional older audiences including “oldest old” (85+ years; Le et al., 2014) participants, older individuals residing in other countries and cultural environments, and older adults with different levels of education, income, previous experience with robots, media usage and exposure, self-efficacy, and physical and cognitive functioning. Such studies should also follow up assimilation processes for longer periods to explore how uses, constraints, and outcomes continue to evolve over time, and compare assimilation of SARs by using additional types of robots, including multi-function vs single-function SARs, stationary vs mobile SARs, and proactive vs reactive SARs.

## Data Availability

The original contributions presented in the study are included in the article/Supplementary material, further inquiries can be directed to the corresponding author.
