# Corrigendum: Assimilation of socially assistive robots by older adults: an interplay of uses, constraints and outcomes

**DOI:** 10.3389/frobt.2024.1438912

**Published:** 2024-06-28

**Authors:** Oded Zafrani, Galit Nimrod, Maya Krakovski, Shikhar Kumar, Simona Bar-Haim, Yael Edan

**Affiliations:** ^1^ Department of Industrial Engineering and Management, Ben-Gurion University of the Negev, Beer-Sheva, Israel; ^2^ Agricultural Biological Cognitive Initiative, Ben-Gurion University of the Negev, Beer-Sheva, Israel; ^3^ Department of Communication Studies, Ben-Gurion University of the Negev, Beer-Sheva, Israel; ^4^ The Center for Multidisciplinary Research in Aging, Ben-Gurion University of the Negev, Beer-Sheva, Israel; ^5^ Department of Physical Therapy, Ben-Gurion University of the Negev, Beer-Sheva, Israel

**Keywords:** acceptance, aging, assimilation, human-robot interaction, older adults, quality evaluation, socially assistive robots, wellbeing

In the published article, there was an error in the article title. Instead of “*Assimilation of socially assistive robots’ by older adults: an interplay of uses, constraints and outcomes*”, it should be “*Assimilation of socially assistive robots by older adults: an interplay of uses, constraints and outcomes*.”

The authors apologize for this error and state that this does not change the scientific conclusions of the article in any way.

